# Modulation of Electronic Availability in g-C_3_N_4_ Using Nickel (II), Manganese (II), and Copper (II) to Enhance the Disinfection and Photocatalytic Properties

**DOI:** 10.3390/molecules29163775

**Published:** 2024-08-09

**Authors:** Angie V. Lasso-Escobar, Elkin Darío C. Castrillon, Jorge Acosta, Sandra Navarro, Estefanía Correa-Penagos, John Rojas, Yenny P. Ávila-Torres

**Affiliations:** 1 Environmental Remediation and Biocatalysis Research Group (GIRAB), Institute of Chemistry, University of Antioquia UdeA, Calle 70 No. 52-21, Medellín 050014, Colombia; vanessa.lasso@udea.edu.co (A.V.L.-E.); elkindariocastellon@gmail.com (E.D.C.C.); jlacosta@utp.edu.co (J.A.); estefania.correa@udea.edu.co (E.C.-P.); jhon.rojas@udea.edu.co (J.R.); 2 Grupo de Investigación Cecoltec, Cecoltec Services, Cra 43 A 18 sur 135, Medellín 050022, Colombia; snavarro@cecoltecservices.com

**Keywords:** coordination compounds, disinfection, degradation, carbon nitride

## Abstract

Carbon nitrides can form coordination compounds or metallic oxides in the presence of transition metals, depending on the reaction conditions. By adjusting the pH to basic levels for mild synthesis with metals, composites like g-C_3_N_4_-M(OH)_x_ (where M represents metals) were obtained for nickel (II) and manganese (II), while copper (II) yielded coordination compounds such as Cu-g-C_3_N_4_. These materials underwent spectroscopic and electrochemical characterization, revealing their photocatalytic potential to generate superoxide anion radicals—a feature consistent across all metals. Notably, the copper coordination compound also produced significant hydroxyl radicals. Leveraging this catalytic advantage, with band gap energy in the visible region, all compounds were activated to disinfect E. coli bacteria, achieving total disinfection with Cu-g-C_3_N_4_. The textural properties influence the catalytic performance, with copper’s stabilization as a coordination compound enabling more efficient activity compared to the other metals. Additionally, the determination of radicals generated under light in the presence of dicloxacillin supported the proposed mechanism and highlighted the potential for degrading organic molecules with this new material, alongside its disinfectant properties.

## 1. Introduction

Carbon nitrides have gained significant importance owing to their high physicochemical stability and low toxicity, making them suitable for applications in photocatalysis for environmental remediation and various emerging technologies [1,2,3]. However, certain thermodynamic properties of g-C_3_N_4_, such as its lipid-soluble character and the potential aggregation of its layers through hydrophobic interactions, along with considerations related to surface geometry, introduce some limitations in its electronic properties. 

Notably, these compounds exhibit a high electronic recombination index, diminishing their capacity to generate electron-hole pairs. Additionally, the positioning of energy levels concerning redox potentials and the proper alignment of energy bands are critical factors influencing their efficiency and photochemical performance [4,5]. Several alternatives currently exist to enhance the optoelectronic properties of g-C_3_N_4_. For instance, the synthesis of heterojunctions has the potential to facilitate the efficient transfer of charge carriers between materials, mitigating undesired recombination and thereby improving the photocatalytic process [6,7,8]. 

Alternatively, the coordination of g-C_3_N_4_ with transition metals as composite or doping can broaden the absorption spectrum, creating active centers within the polymer mesh that facilitate light absorption, promote efficient charge transfer, and consequently increase the generation of electron-hole pairs [9,10,11]. 

**Scheme 1:**
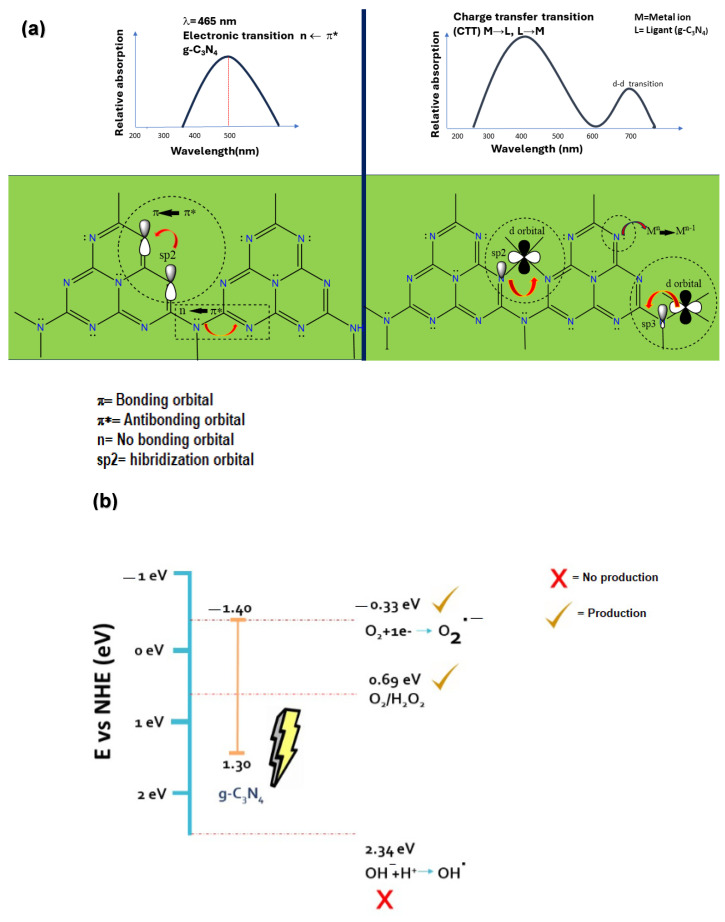
(**a**) Electronic effect of metal ions on electronic properties in g-C_3_N_4_. (**b**) Electronic condition of g-C_3_N_4_ reported vs. generation of ROS.

Coordination with metals introduces new energy levels within the electronic structure of g-C_3_N_4_, influencing the position of electronic states. The stacking of graphitic carbon nitride allows coordination to metal centers through the nitrogen’s lone electron pairs, in contrast to graphene. This can enhance its photocatalytic properties and charge transfer to a metal or an organic molecule. Metals such as Ni, Cu, and Mn, for example, can facilitate charge transfer in the materials, with their ions having the potential to be included in electron-rich cavities on the material’s surface, providing a platform for stabilizing transition metals, even on their surface [12,13,14,15,16], Scheme 1a, Table S1 [13,17,18,19,20,21,22,23,24,25,26,27]. Previous research suggests that amino groups play a role in facilitating the transfer of photogenerated electrons. At the same time, metal ions, owing to their lower work function, can extract photogenerated electrons from semiconductors. In this scenario, coordination doping could potentially result in a synergistic effect between the amino groups and metal ions. 

Moreover, the generation of reactive oxygen species (ROS), such as hydroxyl radicals (^•^OH) and hydrogen peroxide (H_2_O_2_), can be achieved. For instance, Cu is effective in producing ^•^OH, while Ni and Mn can contribute to other ROS species. Consequently, by integrating the generation of ROS with the enhanced photocatalytic properties of the coordinated material, it becomes possible for electron-hole pairs and photogenerated ^•^OH radicals to infiltrate the bacterial cell wall. This intrusion, upon breaking the cell wall, leads to the deterioration and leakage of the cell cytoplasm, ultimately causing bacterial death [28,29,30,31,32]. To generate these species, the electrochemical potentials of the valence and conduction bands must energetically allow the oxidation reactions in the water molecule’s hole and the reduction of oxygen in the conduction band, as shown in Scheme 1b.

Following the previous context, the present study investigated the effect of nickel (II), manganese (II), and copper (II) coordination on the g-C_3_N_4_ template, considering different arrangements for coordinating electron pairs of nitrogen atoms available in the structure and its co-precipitation with metallic hydroxide using soft synthesis. Subsequently, the disinfection activity against *E. coli* 25922 on the surface was evaluated for each of the characterized compounds, and finally, the best material was selected for the degradation of a recalcitrant antibiotic in hospital wastewater (dicloxacillin). Additionally, the improved photocatalytic properties of the copper modification in the presence of dicloxacillin were shown to be correlated, theoretically contributing to the adsorption equilibrium of the system versus the degradation for possible interactions of copper with the matrix and the contaminant.

## 2. Results

### 2.1. Morphology and Spectroscopic Characterization of g-C_3_N_4_ and Modified Materials with Copper (II), Manganese (II), and Nickel (II)

Figure S1 depicts the DRX diffraction patterns for g-C_3_N_4_ and modified products with divalent cations. To consider a fully polymerized tri-s-triazine-based g-C_3_N_4_, a structure was generated starting from the unit cell proposed by Teter and Hemley. In this study, the same reflections were also observed for the tris-triazine-based structure except for a shift towards lower angles [33].

In Figure S2a,b, the condition’s reaction to basic pH evidenced different electronic spectra for each metal transition modification, finding different effects in their relationship with g-C_3_N_4_. Likewise, the lamp identified as adequate for the photocatalytic posterior analyses overlapped the day lamp emission spectrum (Sylvania lamps FT5T8 8500 K (40 cm length) and emitted wavelengths between 400 and 700 nm, as this range warranted the best conditions for interaction for all the materials. The function of the previous information was carried out for the optimization structures for all compounds using BIOVIA Material Studio 2017 (MS2017)—Compass II Forcefield. Energetic parameters were evaluated for structures with two coordination environments: copper (II) modification (bonding to N-aliphatic), manganese (II), and nickel (II) (bonding to N-aromatic) and high hydroxide presence, Figure S3. For hydroxide metallics, the X-ray structures that are reported and compared in Figure S1 were used. Figure S4a–d depicts the morphology and microstructure of the g-C_3_N_4_ samples that were revealed by SEM and TEM. The products investigated using SEM exhibited aggregated morphologies and hydroxide presence in nickel and manganese (II) modifications for layers. The TEM micrographs revealed that the g-C_3_N_4_ has a lamellar, sheet-like structure (Figure S4e), as has been reported previously. However, Ni-g-C_3_N_4_, as shown in Figure S4f, appeared to have aggregated particles, which contained many smaller crystals.

In Table 1, an elemental surface analysis was quantified from the SEM results to establish the nature of the bond to the metal ion in the function of the stoichiometric relationship. Table 2 lists the results of the leaching studies with the detection of ionic chromatography. These results investigated the stability of the compounds in aqueous solution. 

Table 3 reports the PC (Potential Charge) using dynamic scattering light, which showed a residual negative charge for the graphitic nitride. After modification with metal transitions, there were significant effects on surface charge. 

The microstructure of the g-C_3_N_4_ and modified materials was studied via nitrogen-conducting adsorption–desorption experiments, and the results are displayed in Figure S5. All materials exhibited some degree of hysteresis related to a type IV isotherm. An in-depth analysis of these hysteresis indicated that all the materials presented a distinctive, plate-shaped pore system. The pores’ properties, hysteresis degree (%), and fractal dimensions were reported in Table 4.

X-ray photoelectron spectroscopy (XPS) was employed to investigate the surface chemical compositions. The full survey XPS spectrum (Figure S6a) showed the presence of Ni, Mn, and Cu at the first inflection point; it was possible to find the band gap energy values of the modified materials, which were 2.70, 2.09, and 1.50 eV for the Cu-g-C_3_N_4_, Mn-g-C_3_N_4_, and Ni-g-C_3_N_4_, respectively. Compared with the theoretical band gap energy of the g-C_3_N_4_, which is 2.65 eV, the introduction changed the band gap width because it forces the creation of new electronic transitions with the d orbitals of the transition metals and the π orbitals of the carbon nitride [34]. Figure S6b–d depicts the contributions for N1s, O1s, and Mn 2p.

### 2.2. Electrochemical Characterization of g-C_3_N_4_ and Copper (II)-, Manganese (II)-, and Nickel (II)-Modified Materials

**Figure 1:**
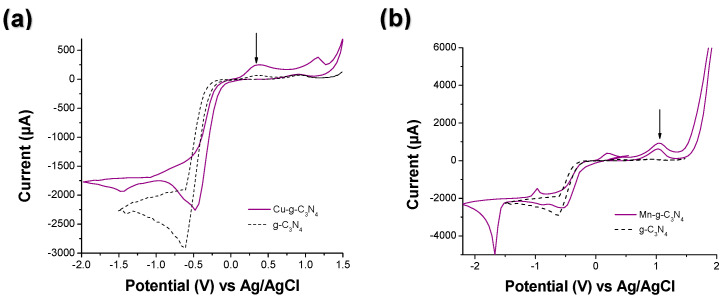

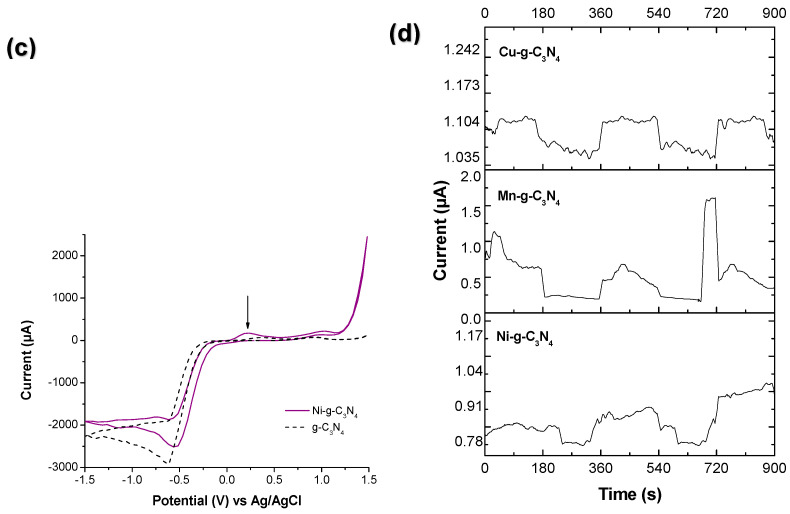
Cyclic voltammetry curves for (**a**) Cu-g-C_3_N_4_, Ni-g-C_3_N_4_, (**b**) Mn-g-C_3_N_4_, and (**c**) Ni-g-C_3_N_4_. Experimental conditions: sweep rate = 20 mV/s; window potential = −1.5 V and 0.0 V, supporting electrolyte: [Na_2_SO_4_] = 0.1 M; and [H_2_O_2_] = 0.01 M. (**d**) Chronoamperometry curves for Cu-g-C_3_N_4_, Mn-g-C_3_N_4_, and Ni-g-C_3_N_4_. Experimental conditions: V = 0.5 V; time = 15 min; supporting electrolyte: [Na_2_SO_4_] = 0.1 M; and [H_2_O_2_] = 0.01 M. The ↓ corresponds to first oxidation process.

Samples were deposited on fluorine-doped tin oxide (FTO) as a transparent substrate, and cyclic voltammetry (CV) diagrams were initially created with light, Figure 1. For the CV measurements, the scanning rate was kept at 20 mV s^−1^. The tests were performed in three cycles between −1.5 V and +1.5 V for Cu-g-C_3_N_4_, Ni-g-C_3_N_4_, and Mn-g-C_3_N_4_, and between −2.2 V and 2.2 V for g-C_3_N_4_, as the supporting electrolyte: [Na_2_SO_4_] = 0.1 M. It was observed that the material exhibited an optical band gap, where the potential of the anodic and cathodic peak shifts with the interaction of light for these materials, demonstrating photoactivity or the presence of a photo response within the visible regions. 

The potential versus current plots of the carbon nitride show that the cathodic peaks shifted in metal-modified materials. It is worth noting that in the voltammogram with copper, copper (II) underwent electrochemical reduction to copper (I), demonstrating the presence of this oxidation state. However, this methodology was not entirely suitable for estimating the position of the valence and conduction bands. For this reason, Electrochemical Impedance Spectroscopy (EIS) measurements using the Mott–Schottky method were depicted to understand this mechanism. 

### 2.3. Photocatalytic Disinfection against E. coli 25922 Using g-C_3_N_4_ and Modified Materials with Copper (II), Manganese (II), and Nickel (II)

**Figure 2:**
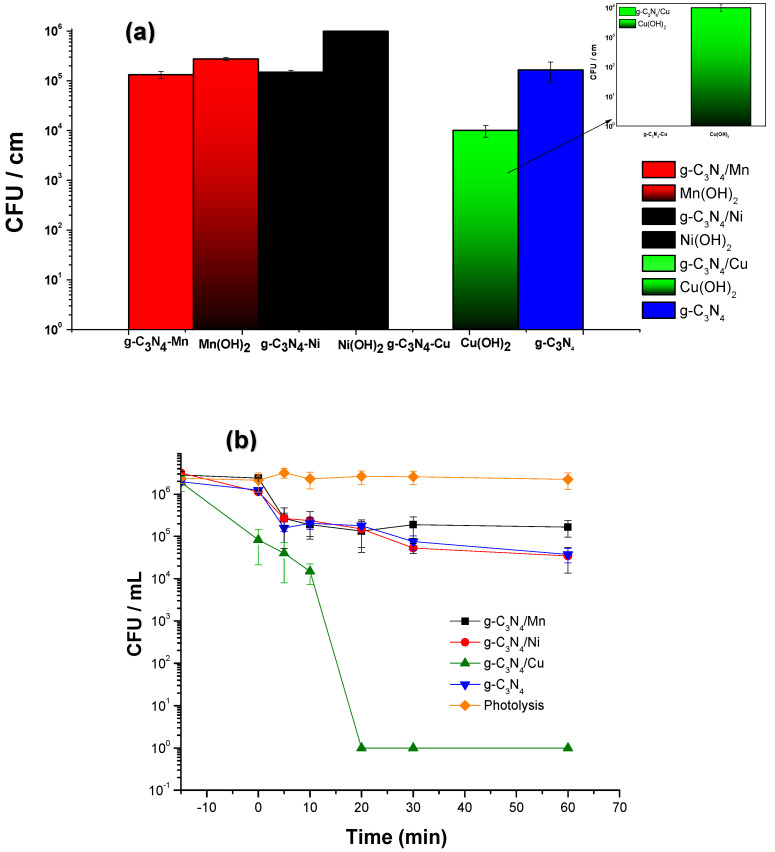
(**a**) Photodisinfection using hydroxides of metal ions versus materials with g-C_3_N_4_ after 20 min of exposure with a Xenon lamp. (**b**) Kinetic disinfection for g-C_3_N_4_ and modified materials with copper, manganese, and nickel against *E. coli* 25922 (Time = −15–60 min).

To discern the contribution of the hydroxides of the respective metals independently, their antimicrobial activity against the E. coli strain was estimated simultaneously with their respective composites consisting of carbon nitrides alternated with metal hydroxides as explained previously, using the best time treatment (20 min). The hydroxide moieties showed antimicrobial activity as follows: Cu(OH)_2_ > Mn(OH)_2_ > Ni(OH)_2_. With a difference between them of 3 Log units, these are not greater than the activity of each of the g-C_3_N_4_, Figure 2a. Figure 2b depicts the disinfection kinetic for g-C_3_N_4_, its modifications with metal transitions, and photolysis control in the range visible. 

### 2.4. Understanding the Disinfection Mechanism of Cu-g-C_3_N_4_

**Figure 3:**
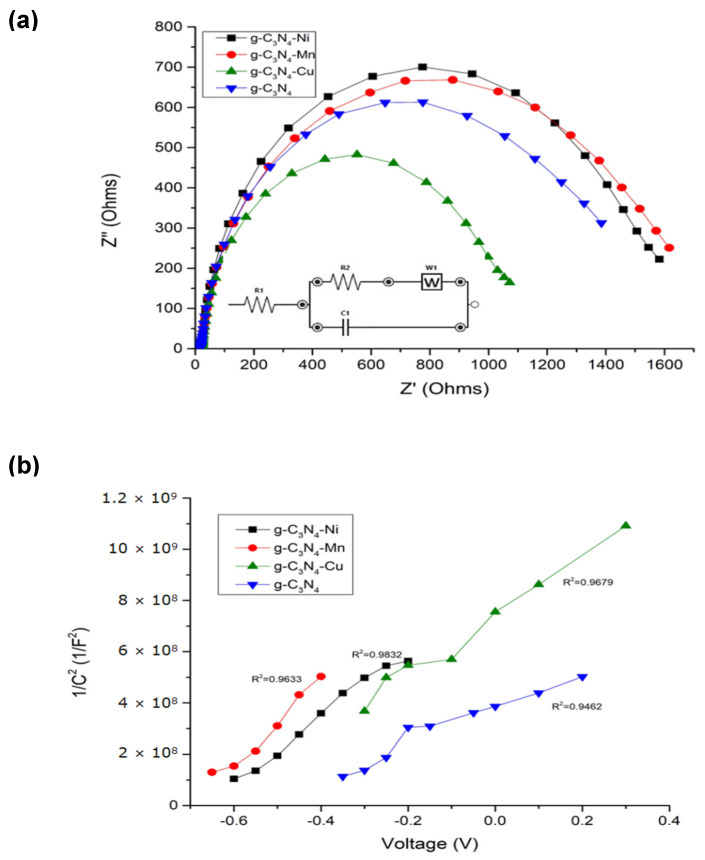

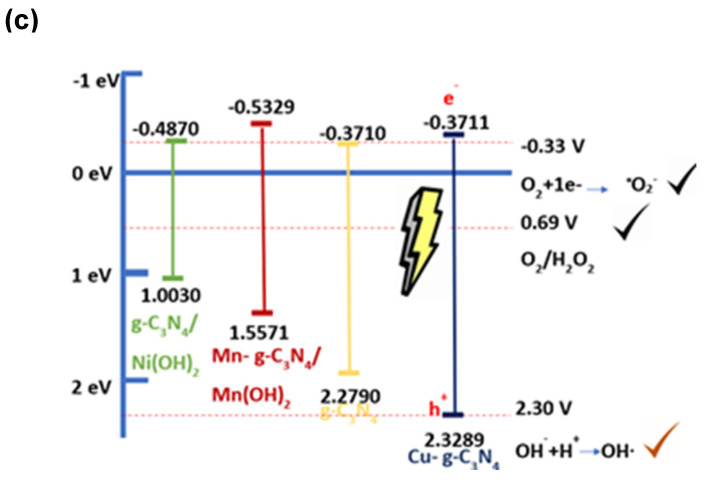
Electrochemical characterization of g- C_3_N_4_ and modified materials with copper (II), manganese (II), and nickel (II), (**a**). Nyquist diagrams, (**b**). Mott–Schottky plot, (**c**). Bands (conduction and valence) diagram construed for authors.

It is particularly useful in the study of semiconducting electrodes to provide information about the charge transfer and separation process. The scans shown in Figure 3 were recorded in the potential window from −1.0 V to 0.5 V (vs. Ag/AgCl) with an increment of 0.05 V at 10 Hz frequency. The principle behind the Mott–Schottky method is based on the relationship between the capacitance of the space charge of a semiconductor electrode and the applied potential. When a positive potential is applied to the semiconductor electrode in an electrolytic solution, a space charge region is established at the semiconductor–electrolyte interface. The variation in capacitance of this region as a function of potential is known as the Mott–Schottky curve. By analyzing this curve, several key pieces of information can be extracted: (i) the position of the flat band potential or conduction band of the semiconductor within the solution, (ii) carrier density in the semiconductor, (iii) the direction of the slope of the curve, indicating whether the carriers are electrons or holes, and (iv) the conduction band and valence band. Figure 3a depicts the Nyquist diagrams and Mott–Schottky plot. The impedance curve is a semi-circular curve that shows that the process of a three-electrode system is controlled by charge transfer. A smaller semi-circular arc of impedance indicates a faster electron transfer rate on the material’s surface and a more probable electrochemical redox reaction; therefore, it indicates a decrease in charge–transfer resistance [35]. Likewise, calculating for Mott–Shottky plots is commonly used to determine the flat band potential of the semiconductor catalysts, and these are shown in Figure 3b. In conclusion, regarding valence and conduction bands associated with impedance study, Figure 3c depicts these electrochemical potentials in relationship with the hydroxyl, anion superoxide formation. 

**Figure 4:**
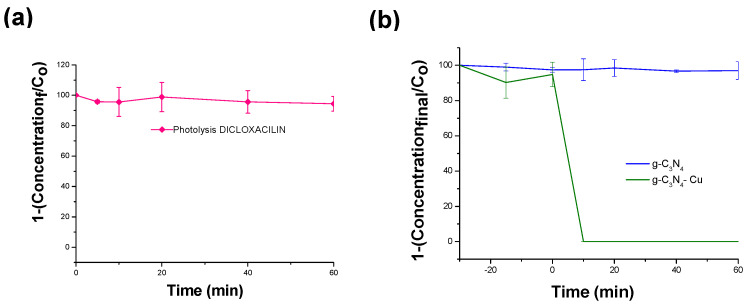

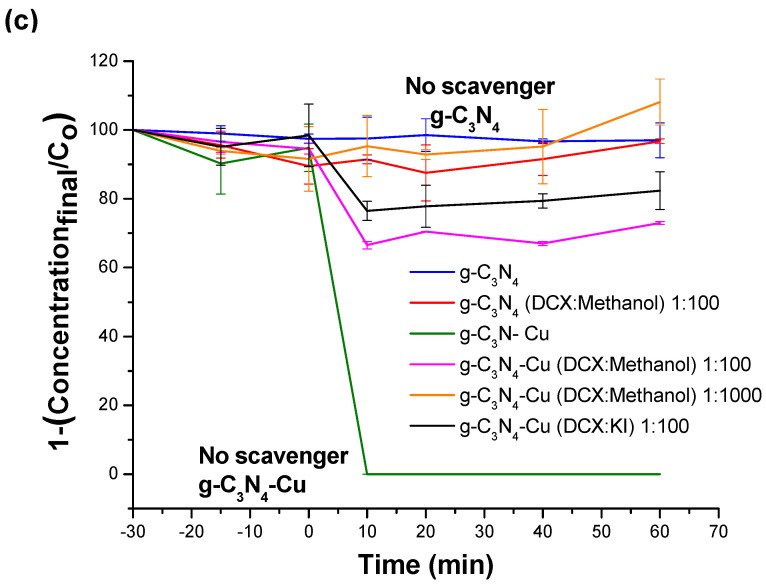
(**a**). Photolysis of DXC to Xenon Lamp, (**b**). Photodegradation using Cu-g-C_3_-N_4_ and g-C_3_-N_4_ with exposure to Xenon lamp, (**c**). Photodegradation using Cu-g-C_3_-N_4_ and g-C_3_-N_4_ with scavengers for ^•^OH radical and h^+^ activation.

To confirm the presence of hydroxyl radicals in Cu-g-C_3_N_4_, as confirmed by electrochemical studies, scavengers like methanol (targeting hydroxyl radicals) and potassium iodide (targeting hole oxidation) were used to understand the mechanism linked to this disinfection property as compared to other materials. However, the methanol disinfection activity is well-known, complicating the interpretation of kinetic disinfection in bacterial studies. Organic molecules such as dicloxacillin are sensitive to reactive oxygen species (ROS) and, therefore, are used to examine the kinetics of degradation and to determine the evolution of ROS; the photolysis control was considered for this molecule, Figure 4a,b. Posteriorly, the scavengers (KI: holes specific; Methanol: ^•^OH specific) were used to reveal the production of the ROS for the best material for the disinfection properties of Cu-g-C_3_N_4_.

**Figure 5:**
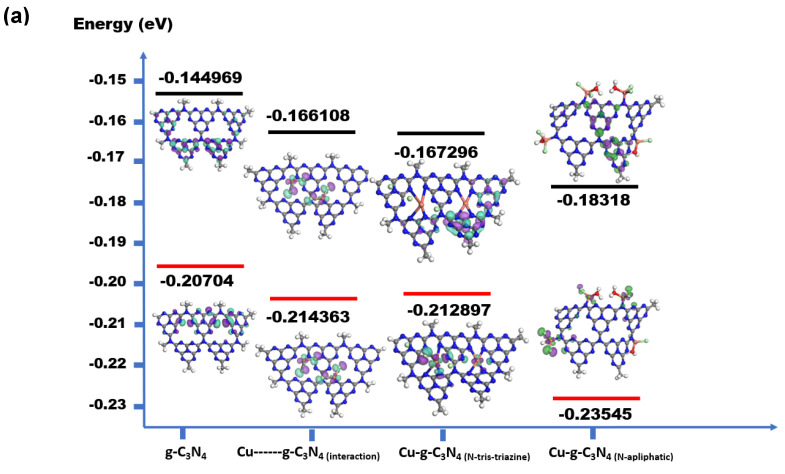

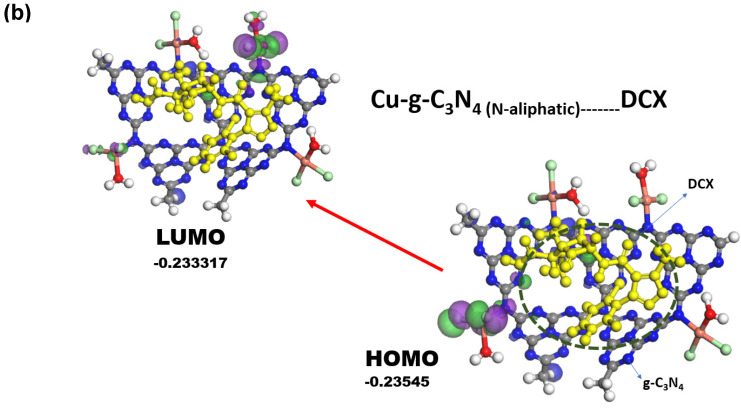
(**a**) Theoretical calculations for a system composed of g-C_3_-N_4_, Cu-g-C_3_-N_4_ (physisorption), CuN-g-C_3_N_4_ (Cu-coordinated N-triazine), and CuNH-g-C_3_N_4_ (Cu-coordinated N-aliphatic). (**b**) CuNH-g-C_3_N_4_ (Cu-coordinated, N-aliphatic-interacting DCX molecule). Color code structure: blue color (nitrogen atom), red (oxygen atom), grey (carbon atom), white (hydrogen atom), purple (negative density charge), green (positive density charge), and dark green (chlorine atom).

This type of interaction with metal ions can modify the local electronic structure, facilitating the transfer of electrons between the orbital metals and the nitrogen atom. As described previously, copper is found around the aliphatic nitrogen located at the periphery of the carbon nitride. To understand if the interaction between the N-triazine and N-aliphatic represents lower energy in the system and low leaching, computational calculations were performed by comparing the structure of nitride with coordinated copper at the metal periphery, around the N-triazines, exerting an electrostatic adsorption interaction, Figure 5a. This resulted in the best structure of copper for interacting with dicloxacillin in the adsorption studies, Figure 5b. 

## 3. Discussion

Figure S1 presents, for instance, the (210) reflection observed at 2θ = 11.5°, (101) at 2θ = 17.54°, and (002) at 2θ = 27.78°, corresponding to the tris-triazine ring. The modifications related to the metal ion depend intrinsically on their ability to coexist in their hydroxylated form at the working basic pH. For example, in the case of nickel, the structure was greatly modified, having a significant contribution from the planes (001) (100) (101) corresponding to Ni(OH)_2_ [36]. Likewise, but to a lesser extent, this phenomenon was observed for copper and manganese. Conversely, the (210) plane corresponded to the orthorhombic reflection, which was associated with the alignment of the aromatic layers. In the case of copper, the plane remains like the g-C_3_N_4_ material, showing a slight distortion in the conformation of the aromatic rings. This suggests that the copper ion has little effect on the planar structure and can be located on the periphery of the nitride template, located in the N-aliphatic fragment. On the other hand, nickel (II) and manganese (II) had a large contribution to the N-N-triazine structure, and, consequently, the relationship between the I_210_/I_002_ planes distorted the aromatic rings of the triazines, which are known to have an AA stacking.

In Figure S2a,b, the Mn-g-C_3_N_4_ spectra presented a highly symmetric D4h coordination environment and a large planar ð electron system around 400 nm. The former gave rise to d-orbital degeneracy or near-degeneracy and the possibility of high spin multiplicity. In this case, the metal dx^2^-y^2^ orbital, which by convention points at the N-N-triazine, is predicted to be quite high in energy [37]. For copper (II) the electronic spectra were very different to homologs with manganese (II) and nickel (II); the band to 440 nm was related to tetrahedral environments [38]. Finally, nickel (II) presented few electronic changes; it suggested modifications in the structure due to the pH and the hydroxyl compounds in the solution. To approach this information, the lamps in the visible region were selected for the posterior analyses.

In Figure S3a–d, the structure with low energy for copper (II) is suggested for Cu-N-aliphatic. In contrast, in the manganese (II) structure, there are moderate contributions of hydroxides, but nickel stability is governed by the hydroxides nickel (II) [39,40]. This preliminary structure allows suggested different electronic properties for all modifications in relationship with carbon nitride.

In Figure S4a–d for copper micrographs, a greater distribution of the metal ion is observed, which is consistent with homogeneous coordination of the material on the periphery of the carbon nitride. Conversely, nickel- and manganese-modified materials showed a metal distribution into spots, which may be attributed to the alternation between metal hydroxides and the carbon nitride template [41]. The TEM images suggest that Ni (OH)_2_ crystals are stacked with the g-C_3_N_4_, as proposed previously. The diffraction pattern shows the characteristic planes of (100), (001), and (101) attributed to nickel hydroxide in the region associated with this structure, Figure S4e,f.

In Table 1, the presence of copper in carbon nitride increases the percentage of oxygen on the surface (EDS analysis), suggesting a prevalent coordination of water molecules from the metal surface and the environment under mild reaction conditions, after the polymerization of urea. The stoichiometric relationship associated with hydroxides on the surface is few, as suggested by these results. These water molecules may complete the coordination sphere of the copper metal ion. In contrast, modification with nickel and manganese shows that the percentage of oxygen increases concerning the metal percentage in a 2:1 stoichiometry (oxygen: metal), suggesting the presence of hydroxides in the systems containing these two metals. Interestingly, for nickel, there was a slight variation in the percentage of carbon and nitrogen as compared to carbon nitride, suggesting that this graphitic structure is alternated with nickel (II) hydroxide. On the other hand, the metal leaching capability in an aqueous solution rendered the Ni-g-C_3_N_4_ and Cu-g-C_3_N_4_ materials fully stable in solution. However, the manganese compound presents moderate leaching, which is above the range put forth by the regulatory agencies (0.12 mg/L) [42], Table 2.

On the other hand, the DLS suggests deprotonation of oxygenated groups on the periphery for g-C_3_N_4_. However, the modification with nickel (II) and manganese (II) followed the hydroxyl group’s surface. Surprisingly, the copper-modified material exhibited a more positive value in the residual potential of the system, which is explained by the metal coordination on the material’s periphery compensating for the innate surface charge of carbon nitride. Likewise, the particle size increases with copper (II) modification, which is related with the disposition surface for g-C_3_N_4_ with a metal ion coordinated in the N-N-aliphatic, which does not limit the electronic effect of N-triazine and its availability, Table 3.

Figure S5, the g-C_3_N_4_, Mn-g-C_3_N_4_, and g-C_3_N_4_-Cu isotherms belonged to the H3-type isotherm, indicating a parallel and narrow plate-shaped pore conformation. Conversely, g-C_3_N_4_-Ni depicted an H4-type isotherm, suggesting a prevalent slit/wedge-shaped pore conformation. Particularly, Ni-g-C_3_N_4_, and to a lesser degree Cu-g-C_3_N_4_, showed a p-stacking of the g-C_3_N_4_ due to the tensile strength effect [43]. The specific surface areas of g-C_3_N_4_, Ni-g-C_3_N_4_, Mn-g-C_3_N_4_, and Cu-g-C_3_N_4_ were estimated to be 52.7 m^2^ g^−1^, 80.9 m^2^ g^−1^, 51.4 m^2^ g^−1^, and 47.4 m^2^ g^−1^ respectively. The presence of manganese had a negligible effect on the surface area of the graphitic carbon nitride, as previously mentioned. On the contrary, the surface area of nickel carbon nitride was high due to the presence of hydroxide moieties alternated on the surface, disrupting the 3D structure of the graphitic nitride. This effect was also noticed on the surface roughness as expressed by the fractal dimension, evidencing the presence of co-structures on surfaces such as hydroxides (Table 4). Likewise, the copper-modified materials’ roughness was comparable to that of carbon nitride, presuming the limited presence of surface hydroxides forming a coordination of the metallic center at the periphery of the structure. In the case of copper, the charge potential was affected by the coordination of the metal center, resulting in a large particle size. All materials were characterized as having a prevalent mesoporous system. However, the presence of nickel metal decreased the degree of mesoporosity and increased the micropore degree and width. This is explained by the presence of nickel hydroxides in the material. On the other hand, copper and manganese ions slightly increased the degree of microporosity since these metals coordinate the free electron pairs of nitrogen.

Figure S6 illustrates peaks corresponding to N1’s and O1’s orbitals within the 200 and 400 eV range. In the case of Cu-g-C_3_N_4_, the peaks observed at 400.4, 398.9, and 399.8 eV can be assigned to the bridging N atoms in N(C)_3_ groups, and the sp^2^-hybridized nitrogen triazine rings of C-N=C and the Cu-NH bond respectively, indicating that the Cu forms a coordination complex with the final amines of the carbon nitride network. Conversely, the Ni-g-C_3_N_4_ and g-C_3_N_4_ peaks at N1s at 401.1 eV suggest the presence of free amino groups (C-N-H) present in the material, suggesting that the formation of the coordination complex of these metals occurs in the triazine rings and 398.3 eV, Figure S6b [44]. From these results, we found the location of the metal in the g-C_3_N_4_ network. These findings are consistent with the XRD pattern, indicating a tris-triazine structure in g-C_3_N_4_, with metal ions interacting with its amine groups. Specifically, copper tends to interact with nitrogen aliphatic regions, as evidenced by XPS showing a greater exposure of triazine groups on the surface. In contrast, manganese ions interact with triazine groups, leading to the exposure of amine aliphatic groups. The O1’s orbital demonstrates the influence of metal centers on g-C_3_N_4_, resulting in an energy shift from 531.16 eV to 534.07 eV for copper with water molecules and hydroxide contributions, Figure S6c. For the Mn 2p orbital, we found two contributions at 641 and 645 eV [45] corresponding to Mn-N and Mn-O, suggesting Mn(OH)_2_ and M-N-triazine, Figure S6d.

Figure 1 shows CV results for all the modified materials and bare g-C_3_N_4_. All measurements were conducted at pH 5.4. According to the Pourbaix diagram for Cu, the main species at work pH is Cu(II). In Figure 1a, there is a reduction peak at 0.33 V associated with the oxidation of Cu (I) to Cu (II), and a cathodic peak at −1.44 attributed to the reduction of Cu (II) to Cu (I). Similar peaks in the CV of Mn-g-C_3_N_4_ (Figure 1b) and Ni-g-C_3_N_4_ (Figure 1c), specifically, anodic peaks at 1.03 V and 0.21 V, are linked to the oxidation of Mn (II) to Mn (IV) and Ni (II) to Ni (III), respectively, according to their Pourbaix diagram. Additionally, cathodic peaks at −0.97 V and −0.73 V are associated with the reduction of the metal species [46,47,48,49]. In Figure 1d, the Cu-gC_3_N_4_ photocurrent carriers were recyclable and stable under the on–off cycles, and it had the highest photocurrent. In contrast, for Mn-g-C_3_N_4_ and Ni-g-C_3_N_4_, the photocurrent changed with every on–off switch. As discussed in the previous chapter, the Cu dispersion over the g-C_3_N_4_ template was more homogeneous than in Mn-g-C_3_N_4_ and Ni-g-C_3_N_4_, and the presence of hydroxides affects the flow of the photocarriers over the photocatalyst surface, thereby altering the behavior of these materials.

From the spectroscopic, textural, and electrochemical characterization of new materials under soft synthesis, the disinfection against *E. coli* was determined. Figure 2 shows that the activity of the nitride structures is independent of that of the metal hydroxide, probably given that the generation of ROS in the interaction of g-C_3_N_4_ with the metal is more effective in oxidation processes. The Cu-g-C_3_N_4_ performed a complete disinfection concerning g-C_3_N_4_ and modified materials with manganese and nickel (II). Therefore, Cu in terms of electrical conductivity is more effective than Mn and Ni. This phenomenon is explained by the ability of a metal to participate in redox reactions and release electrons. Copper can be present in ionic forms, such as Cu (II), which can effectively participate in redox reactions and induce the formation of ROS [50]. In contrast, nickel and manganese tend to form more stable oxidation states which are not as likely to participate in redox reactions that generate ROS. Thus, when a metallic ion is integrated into a carbon nitride network, the nitrogen atoms can act as electron-pair donors to form coordination bonds with the metallic ion. This type of interaction can modify the local electronic structure, facilitating the electron transfer between the orbital metals and the nitrogen atom. The metallic ion in carbon nitride serves as a catalyst and provides active sites on the surface for oxidation reactions, these sites may have available electrons in their electronic structure. In the presence of molecular oxygen (O_2_), this structure adsorbs oxygen molecules on the surface [51]. Electrons from the orbital metal can be transferred to oxygen: this electron transfer can occur due to the electrical potential difference between the metal ionic and the adsorbed species. Adsorbed oxygen captures electrons that could reduce them to superoxide species (O_2_^•−^) [52]. The nonbonding orbitals, also called nonbonding electron pairs, can donate electrons without breaking coordination bonds. In other cases, the electronic distribution can change without breaking coordination bonds through the phenomenon of resonance. Electrons can “move” between different positions without changing the connectivity of the coordination bond [53]. On the other hand, the carbons of the triazine ring may play a significant role in donating electrons to the dopant metal. Triazine is a molecule that contains nitrogen and carbon atoms in a heterocyclic ring. Electrons from the carbon atoms in the ring can participate in the transfer of electrons through sigma bonds, stabilizing the structure, and the stability of the ring can influence the donation of electrons to the metal ion [54]. The sp^2^ hybridization gives rise to an unhybridized p-orbital perpendicular to the plane of the triazine ring. This unhybridized p orbital may contain π electrons, which are more available to participate in electron donation processes. Thus, the presence of π electrons in sp^2^ hybridization may allow the ring carbon atoms to participate in redox reactions, since these electrons can be more easily transferred to other chemical species including ionic metals.

Figure 3a,b is used to understand the nature and origin of ROS species with the materials that are semiconductors. Figure 3a shows that Cu- g-C_3_N_4_ has the lowest impedance resistance. This indicates the fastest electron transfer rate, enhancing the separation efficiency of photogenerated carriers. The transfer of charges across the interface between the semiconductor and the solution becomes easier, leading to an increase in the photocurrent and a better chemical catalytic performance [55] as compared to the other three materials [56]. The curves of g-C_3_N_4_, Cu-g-C_3_N_4_, Ni-g-C_3_N_4_, and Mn-g-C_3_N_4_-Cu are depicted in Figure 3b. The electrochemical system can be described by the Randles circuit (inset in Figure 3a), where R1 is the resistance owing to the passage of electrons between the surface of the material to the reference electrode, R2 is due to the charge transfer resistance of the redox couple, W1 is denoted as the Warburg impedance that takes into account diffusion of electroactive species towards the electrode, and C1 corresponded to the non-faradaic capacitance of the g-C_3_N_4_. The flat band potentials were −0.49697, −0.53287, −0.37109, and −0.37105 eV for Ni-g-C_3_N_4_, Mn-g-C_3_N_3_, Cu-g-C_3_N_4_, and g-C_3_N_4_, respectively. It can be observed that the introduction of the transition metal as a complex into the g-C_3_N_4_ framework modified the position of the conduction band. From the thermodynamics standpoint, a more negative conduction band position suggests a major reduction potential. This is translated into a higher production of superoxide anion radical (−0.33 eV) [57].

Knowing the band gap and the conduction band of the materials, it can be calculated the valence bands which were 1.0030, 1.5571, 2.3289, and 2.2790 eV for the Ni-g-C_3_N_4_, Mn-g-C_3_N_4_, Cu-g-C_3_N_4_, and g-C_3_N_4_, respectively. According to the previous results, it can be inferred that the oxidative potential is not enough to generate hydroxyl radicals (2.30 eV), even with the addition of the transition metal in the g-C_3_N_4_, manganese, and nickel systems, as is shown in Figure 3c. However, the valence band for band Cu-g-C_3_N_4_ was enough to produce hydroxyl radicals showing the capacity of all materials to generate superoxide anion radical, which is an ROS with a high redox potential for degrading membrane microorganisms.

In the same context, the dicloxacillin (DCX) molecule was used against scavengers to verify the ROS production. This molecule exhibited minimal degradation through photolysis within a 60 min timeframe, Figure 4a [58]. Figure 4b demonstrates that the adsorption of DCX is reduced when utilizing g-C_3_N_4_ and its copper-modified counterpart. DCX, when at a circumneutral pH, presents a deprotonated carboxylic group, resulting in reduced adsorption owing to the negative potential charge (PC) of the materials. Light exposure significantly influences degradation, with full degradation occurring around 10 min, possibly due to light-activated reactive oxygen species (ROS). To explore the relationship between ROS production, specifically ^•^OH radical and h^+^ activation, scavenger experiments with methanol and potassium iodide (KI) were conducted in the presence of DCX. The degradation kinetics were hindered in the presence of high concentrations of methanol, which acted as a scavenger for DCX degradation. Conversely, KI blocked approximately 15% of the degradation process, indicating direct oxidation by h^+^, Figure 4c. These findings are consistent with the positions observed in the valence and conduction bands obtained from the Mott–Schottky plot. Overall, these results suggest that ^•^OH radicals and h^+^ play significant roles in both disinfection and degradation processes.

Finally, to comprehensively investigate the stability of the copper compound, theoretical calculations were carried out, as shown in Figure 5a. The HOMO with low energy corresponds with metal ions coordinated to nitrogen aliphatic, as was suggested in the spectroscopic characterization. The metal ion presence in the environment of carbon nitride decreased energy levels and compensated for the charge-negative surface. The geometry around copper is suggested to have a tetrahedral geometry, which is congruent with electronic spectra in the solid state [59]. Additionally, it has low stability for a structure with Cu-N-tris triazine. The interaction of the DXC with Cu-g-C_3_N_4_ coordinated the N- aliphatic can be explained as the DCX interacting with de sp^2^-hybridization to increase the active places for the catalyst, Figure 5b.

## 4. Materials and Methods

### 4.1. Reagents

Dicloxacillin (DCX) was provided by Sinopharm Laboratories. Urea was provided by Analytical Standards M&B Laboratory Chemicals (Cape Town, South Africa). Nickel chloride hexahydrate and cupric chloride dihydrate were provided by Loba Chemie Laboratory Reagents (Colaba, India) and Fine Chemicals (Cape Town, South Africa). Manganous chloride tetrahydrate was provided by Baker (Melbourne, Australia). Reagent sodium hydroxide pellets, fluorine-doped tin oxide sheets, perchloric acid, sodium sulfate, hydrogen peroxide, formic acid, and acetonitrile were provided by Merk (Darmstadt, Germany). Nitrogen liquid was purchased from Genex (Sydney, Australia).

### 4.2. Modification of g-C_3_N_4_ Synthesis

Bulk g-C_3_N_4_ was prepared by pyrolyzing urea. Urea (10.0 g) was heated to 550 °C in an atmosphere-controlled oven at a heating rate of 100 °C h^−1^. This pyrolysis was conducted for 3 h, followed by cooling until reaching room temperature. The resulting powder was purified with water and dried at 80 °C.

Modified g-C_3_N_4_ were prepared in a reflux system. Briefly, 0.067 g of the metal salt (copper, nickel, or manganese) was diluted in 50 mL of distilled water and was put in reflux for 0.75 h at 100 °C. Subsequently, 0.2 g of g-C_3_N_4_ was added to the reflux system and allowed to react for 1 h. The reaction mixture was allowed to cool down and the pH was adjusted with concentrated NaOH equimolar until reaching a pH of 10. Finally, it was filtered by gravity and allowed to dry passed through paper (75 μm).

### 4.3. Physicochemical and Spectroscopic Characterization

The catalyst surface morphology was observed by Transmission Electron Microscopy (TEM) using an S-4800 HITACHI at an acceleration voltage of 160 kV (Hitachi, Tokyo, Japan). The crystal structures were analyzed using an X-ray powder diffractometer (Model XPert pro-MPD, Medellín, Colombia). Copper X-ray diffraction (XRD) patterns were collected between 5° and 80° at a scan rate of 4° min^−1^ and an incident wavelength of 0.15406 nm (Cu-Kα). X-ray photoelectron spectroscopy (XPS) patterns were recorded using a PHI5300 Software ESCALAB system SID-10148252, LAXPS software with Mg Kα radiation. Pore size distribution and specific surface areas were obtained using Quantasorb equipment (Nova 1200e, Quanta chrome, Boynton Beach, FL, USA) employing nitrogen gas as adsorbate. The sorption isotherms were determined at relative pressures from 0.03 to 0.95. Dynamic Light Scattering (DLS) (ISO13321) range 0.3 nm–10 mm, humidity via. DRX AERIS High score. Scanning Electron Microscopy (ASTM E1508-12/ASTM E 766 14, 2019, M Committee E04 on Metallography, and is the direct responsibility of Subcommittee E04.11 on X-ray and Electron Metallography, USP<1181> [59]. The solid-state diffuse reflectance spectrum and the solution phase electronic spectrum were recorded on a Shimadzu UV-2401 PC spectrophotometer (Shimadzu, Tokyo, Japan) Agilent 8453 diode array spectrophotometer (Agilent, Santa Clara, CA, USA), respectively.

### 4.4. Electrochemical Characterization

Electrochemical Impedance Spectroscopy (EIS) and Cyclic voltammetry (CV) were performed using a PalmSens5, 2004–2022 Palm Sens BV Version 5.9.4206 Build 30281t electrochemical station coupled with a conventional three-electrode system composed of a working electrode holding the material, a counter electrode of Ir, and a reference electrode of Ag/AgCl/KCl under the Xenon lamps (60 W). Each working electrode was prepared using the impregnation method on FTO-coated glass slides (30 mm × 20 mm × 2 mm size) obtained from a commercial supplier (TechInstro, Nagpur, India). Before each experiment, the FTO sheets were soaked with HOCl_4_ at (1 × 10^−3^ M) for 1 h. Approximately, 0.2 g of each material (g-C_3_N_4_, Ni-g-C_3_N_4_-, Mn-g-C_3_N_4_ or Cu-g-C_3_N_4_) was suspended in 10 mL of water, and the FTO sheet was immersed in each suspension for 30 s, followed by drying on a spin-coater. This process was repeated five times to get a complete FTO coating. The FTO-coated sheet was then calcinated in a gas control oven at 500 °C for 1 h. The latter process was repeated twice to guarantee a complete material deposition on FTO sheets. Mott–Schottky analyses were performed at a frequency of 10 Hz using an amplitude of 10 mV at several potentials. To the electrolyte (Na_2_SO_4_) was added H_2_O_2_ with a finality of oxidizing the hole.

### 4.5. Reaction System

The photocatalytic processes were carried out in a homemade aluminum reflective reactor containing four sunlight lamps (Sylvania FT5T8 8500 K 40 cm length, Medellín, Colombia) that emitted wavelengths between 400 and 700 nm. The photocatalytic processes were conducted at 60 W of light power. Antibiotic solutions (50 mL) were placed in beakers under constant stirring and samples were periodically taken for the analyses.

### 4.6. E. coli 25922 Photodisinfection

Inoculation process for the *E. coli* ATCC 25922 strain: Before each experiment, the bacterial growth culture medium was prepared by dispersing 23.5 g of HIMEDIA Plate Count Agar (PCA) in 1L of distilled millipore water. This PCA medium was dissolved, heated to boiling, and sterilized by autoclaving at 15 psi of pressure for 20 min. Subsequently, the medium was cooled to 57 ± 2 °C, and 19 ± 1 mL portions were poured into 100 × 15 mm sterile Petri dishes. Immediately, the culture medium underwent sterilization under UV radiation for 10 min.

The *E. coli* ATCC 25922 bacterial strain utilized in this study is part of the microbiological collection of the Research Group in *Basic and Applied Microbiology* (MICROBA). A stock suspension of 5 × 10^8^ Forming Colony Units (FCU)/mL was prepared as follows: *E. coli* (ATCC 25922) was retrieved from glycerol at −20 °C using a loop. It was then streaked on agar contained in a petri dish, which was incubated for 18 h at 37 °C. Afterward, one colony was suspended in 5 mL of H_2_O and stirred using a vortex mixer. This dispersion was analyzed at 540 nm using a Shimadzu spectrophotometer at 568 nm. A concentration of 10^8^ FCU/mL was adjusted to an absorbance of 0.7.

A closed photoreaction system with temperature control composed of a 2.2 kW Xenon lamp and two vortex systems equipped with a petri dish was used. This system allows for a high interaction with light and materials. In all cases, under dark conditions, 150 mL of distilled water was mixed with 1 mL of the previously described bacterial suspension to obtain a final concentration of 5 × 10^6^ CFU/mL, which was confirmed experimentally. Then, 150 mg/L of the chosen photocatalyst was added and stirred at 250 rpm. The lamp was turned on 15 min later, and a second sample was taken. From that time onward, sampling was conducted at various intervals (t = −15, 0, 5, 10, 20, 30, 60 min). The samples were diluted (1/10) successively in saline solution when necessary (10^5^–10^2^ CFU microorganisms). In all cases, the extracted bacterial suspensions were spread on plate count agar and incubated for 24 h. The number of CFUs was manually counted, and the CFU/mL was calculated. Each experiment was conducted at least in duplicate.

### 4.7. Dicloxacillin Degradation

Quantitative analyses of dicloxacillin were carried out using a UHPLC, Thermoscientific Dionex UltiMate 3000 instrument, equipped with an Acclaim 120 RP C18 column (5 μm, 4.6 × 150 mm) and a photodiode array detector (Thermo Fisher Scientific, Waltham, MA, USA). The wavelength of detection was 270 nm and the injection volume was 20 μL. The mobile phase was composed of a mixture of formic acid (10 mmol L^−1^, pH 3.0) and acetonitrile at a 50:50 ratio, 0.7 mL min^−1^).

To identify the metals’ leaching, ionic chromatography was carried out with a brochure: MagIC Net and column Metro A. The injection volume was 10 μL and mixtures of nitric acid (2 mmol L^−1^)/oxalic acid (0.5 mmol L^−1^) were used as mobile phase, conductivity detectors.

### 4.8. Theoretical Approximations of g-C_3_N_4_ and Modified Materials with Copper (II)

Computational modeling was carried out to get some information about the interaction of carbonous material with dicloxacillin and the calculations of density function theory parameters (E_LUMO_, and E_HUMO_). Starting from the experimental structural data within the BIOVIA Material Studio 2017 (MS2017) that was obtained using Compass II Forcefield [60], we created the Cu-g C_3_N_4_ for the function of spectroscopic characterization. A solid solution using water as a solvent at 100 K was considered. The module adsorption locator included within the commercial modeling software 7.0 MS2017, 2020 was used for the computational studies. BIOVIA Forcite is an advanced classical molecular mechanic tool, designed to work with a wide range of force fields, allowing for fast energy calculations and reliable geometry optimizations of molecules and periodic systems. In our study, Forcite was used to optimize the geometry of DXC and g-C_3_N_4_. In this sense, the BIOVIA adsorption location module was employed, which is based on simulated annealing, a metaheuristic algorithm for locating a good approximation to the global minimum of a given function of a large search space. This allows for the identification of the possible adsorption configurations by carrying out Monte Carlo searches of the configurational space of the selected surface–model molecular systems, as the temperature is slowly decreased. To identify additional local minimum energy, the process was repeated several times.

## 5. Conclusions

The availability of the tris-triazine template to be photoactivated and transport electrons from the valence band to the conduction band was mediated by the availability of π-unsaturation. Manganese- and nickel-modified materials contained a M-g-C_3_N_4_/M(OH)_2_ composite which stabilized and blocked the active sites and prevented light from penetrating the carbon nitride template, affecting disinfection and drug degradation. However, in the copper-modified material Cu-g-C_3_N_4_ the stabilization of an electronic transition corresponded to a D4h environment around the metal center, with interaction in N-aliphatic. Copper-modified materials were evenly distributed due to the availability of bonds in target sections such as the periphery. This slight modification demonstrated that the copper compound can generate hydroxyl radicals and radical anion superoxide, different from other modifications. The radical hydroxyl can be generated in the h^+^ as was demonstrated with the KI scavenger. The material g-C_3_N_4_ modified with copper (II) was an excellent photocatalyst for disinfecting *E. coli* but also was able to degrade dicloxacillin in the presence of light. It offers another opportunity for this material in the fields of degradation of organic molecules. Definitively, the hydroxides in the structures modified with the transition metals do not make important contributions to the disinfection against this microorganism; their role may be structural. It wass concluded that a transition metal coordinated in the N-aliphatic of carbon nitride allows a better electron transfer for the generation of ROS. Conversely, interaction with N-tris triazine can hinder interaction. This soft synthesis opens the opportunity to explore new transition metals by interacting with carbon nitride to degrade organic molecules and enrich disinfection processes.

## Figures and Tables

**Table 1:** Elemental surface analysis with SEM–EDS.

Material	Theoretical Metal Amount (%)	Metal (%)	Carbon (%)	Nitrogen (%)	Oxygen (%)
g-C_3_N_4_	---	---	47.79	46.32	5.31
Ni-g-C_3_N_4_	8.23	4.89	42.36	41.42	11.1
Mn-g-C_3_N_4_	9.25	7.33	41.83	36.4	13.87
Cu-g-C_3_N_4_	12.42	15.09	31.7	39.63	13.24

**Table 2:** Aqueous solution stability (ionic chromatographic detection).

Sample	Concentration (ppm)
Ni-g-C_3_N_4_	0
Mn-g-C_3_N_4_	0.572
Cu-g-C_3_N_4_	0

**Table 3:** Z-average (nm) particle size and PC for g-C_3_N_4_ and modified materials.

	g-C_3_N_4_	Cu-g-C_3_N_4_	Ni-g-C_3_N_4_	Mn-g-C_3_N_4_
z-average (nm)	2561	3714	2257	1309
PC	−27.71	−14.98	−21.94	−29.64

**Table 4:** Pore properties in the adsorption of N_2_.

Material	Pore Volume (cm^3^/g)		Pore Amount (%)		Pore Width (Å)		Hysteresis Degree (%)	Fractal Dimension
	Micropore	Mesopore	Micropore	Mesopore	Micropore	Mesopore		
g-C_3_N_4_	0.023	0.103	13.5	86.5	10.1	51.1	1.3	2.47
Ni-g-C_3_N_4_	0.037	0.078	25.5	74.5	12.1	51.1	24.4	2.58
Mn-g C_3_N_4_	0.015	0.157	7.1	92.9	12.7	51.1	4.2	2.42
Cu-g-C_3_N_4_	0.013	0.120	7.8	92.2	12.1	53.4	9.8	2.48

## Data Availability

The original contributions presented in the study are included in the article/Supplementary Material, further inquiries can be directed to the corresponding author/s.

## Supplementary Materials

The following supporting information can be downloaded at: https://www.mdpi.com/article/10.3390/molecules29163775/s1. Figure S1. DRX patterns for g-C_3_N_4_ and modified materials with transition metals, the red lines (Mn(OH)_2_ crystallographic planes reported), black lines (Ni(OH)_2_ crystallographic planes reported), green (Cu(OH)_2_ crystallographic planes reported), Figure S2. The absorption spectrum of (a) Cu-g-C_3_N_4_, g-C_3_N_4,_ and emission spectra of the daylight lamp (red line), (b) Ni-g-C_3_N_4_ and Mn-g-C_3_N_4_ spectrum, Figure S3. Proposed structures for (a) g-C_3_N_4_ and modified products with (b) copper (II), (c)manganese (II), and (d) nickel (II), Figure S4. SEM images of (a) g-C_3_N_4_ (b) Cu-g-C_3_N_4_, (c) Mn-g-C_3_N_4_, (d)) Ni-g-C_3_N_4_, (e) TEM images of g-C_3_N_4_ and Ni-g-C_3_N, and (f) diffraction pattern of Ni-g-C_3_N_4,_ Figure S5. N_2_ adsorption-desorption isotherms of g-C_3_N_4_ and modified materials, Table S1. Electronic properties and application in 2D- 2D structures and Metal-g-C_3_N_4_, Figure S6. XPS spectra for modified g-C_3_N_4_ materials, (a) Survey XPS for g-C_3_N_4_ modifications, (b) High-resolution N 1s orbital contributions, (c) High-resolution O 1s orbital contributions, (d) High-resolution Mn 2p orbital contributions.
