# Supplementary material for: Modulation of Electronic Availability in g-C3N4 Using Nickel (II), Manganese (II), and Copper (II) to Enhance the Disinfection and Photocatalytic Properties
Source: Molecules. 2024 Aug 9;29(16):3775. doi: 10.3390/molecules29163775 (PMC11356843; doi:10.3390/molecules29163775)
Supplement: Supplementary file 1 [file molecules-29-03775-s001.zip › molecules-3112265-supplementary.pdf]

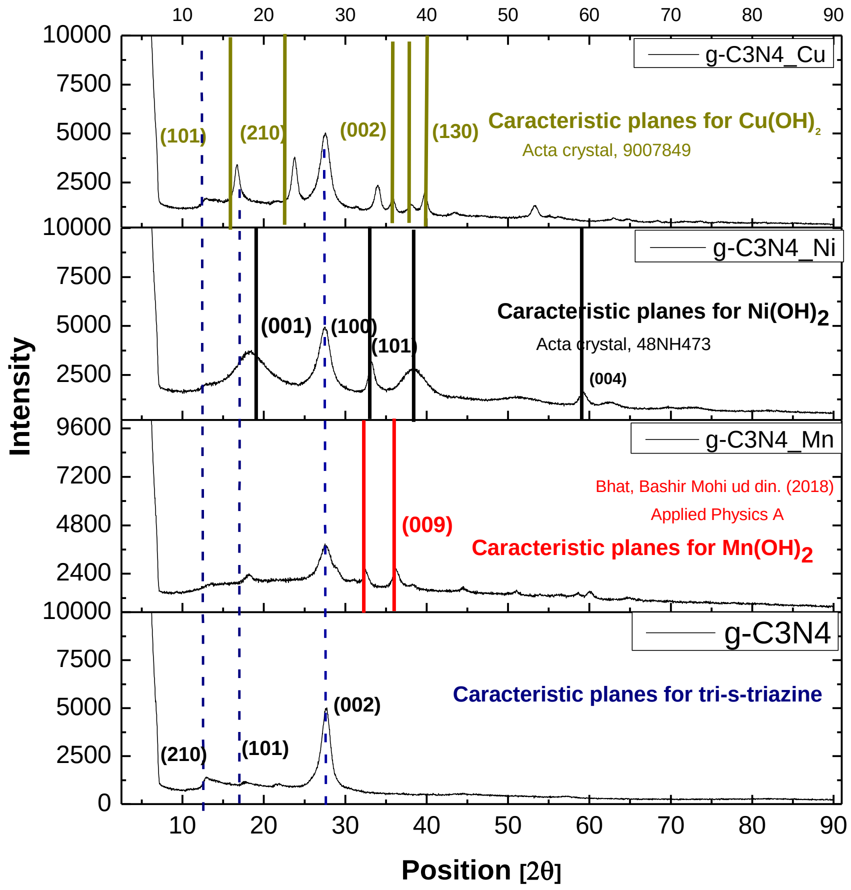

Figure S1. DRX patterns for g-C_3N_4 and modified materials with transition metals, the red lines ($Mn(OH)_2$ crystallographic planes reported), black lines ($Ni(OH)_2$ crystallographic planes reported), green ($Cu(OH)_2$ crystallographic planes reported).

(a)

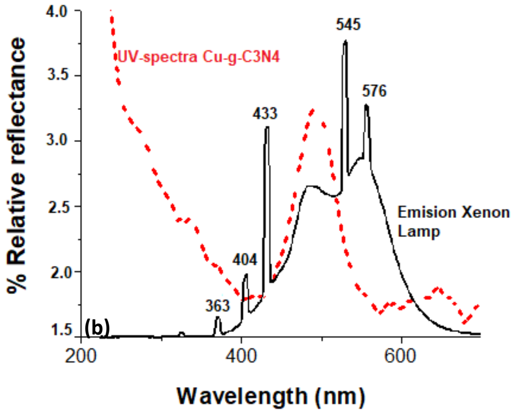

(b)

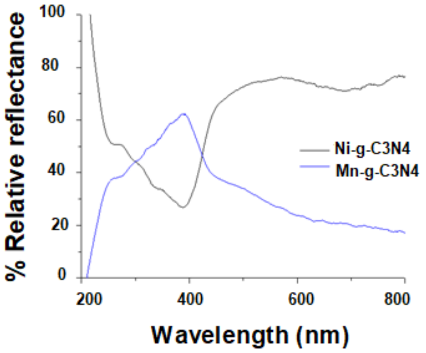

Figure S2. The absorption spectrum of (**a**) Cu-g-C_3N_4, g-C_3N_4, and emission spectra of the daylight lamp (red line), (**b**) Ni-g-C_3N_4 and Mn-g-C_3N_4 spectrum.

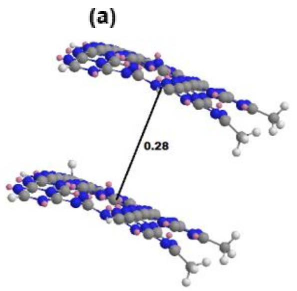

g-C_3N_4 structure
E=638.38 Kcal/mol.

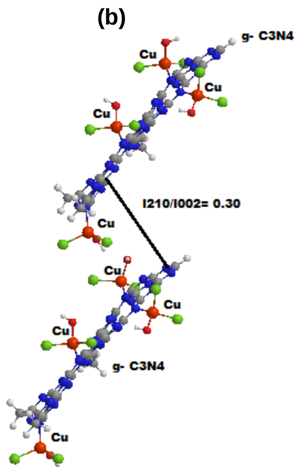

Cu-g-C_3N_4 structure
E=661.1234 Kcal/mol.

(c)

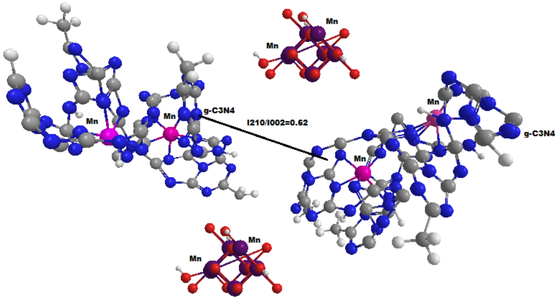

Mn-g-C_3N_4 structure.

(d)

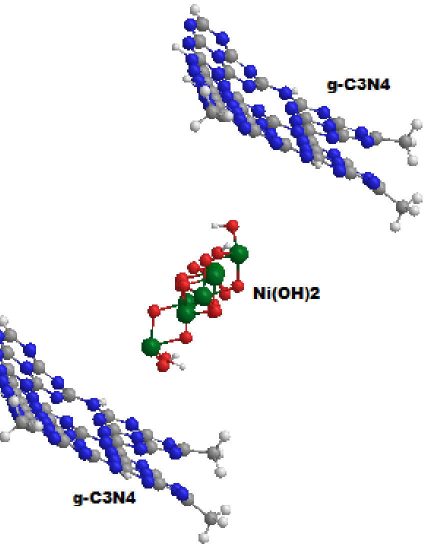

Ni- g-C_3N_4 structure. *E=959.4 Kcal/mol*

Figure S3. Proposed structures for (a) g-C_3N_4 and modified products with (b) copper (II), (c)manganese (II), and (d) nickel (II).

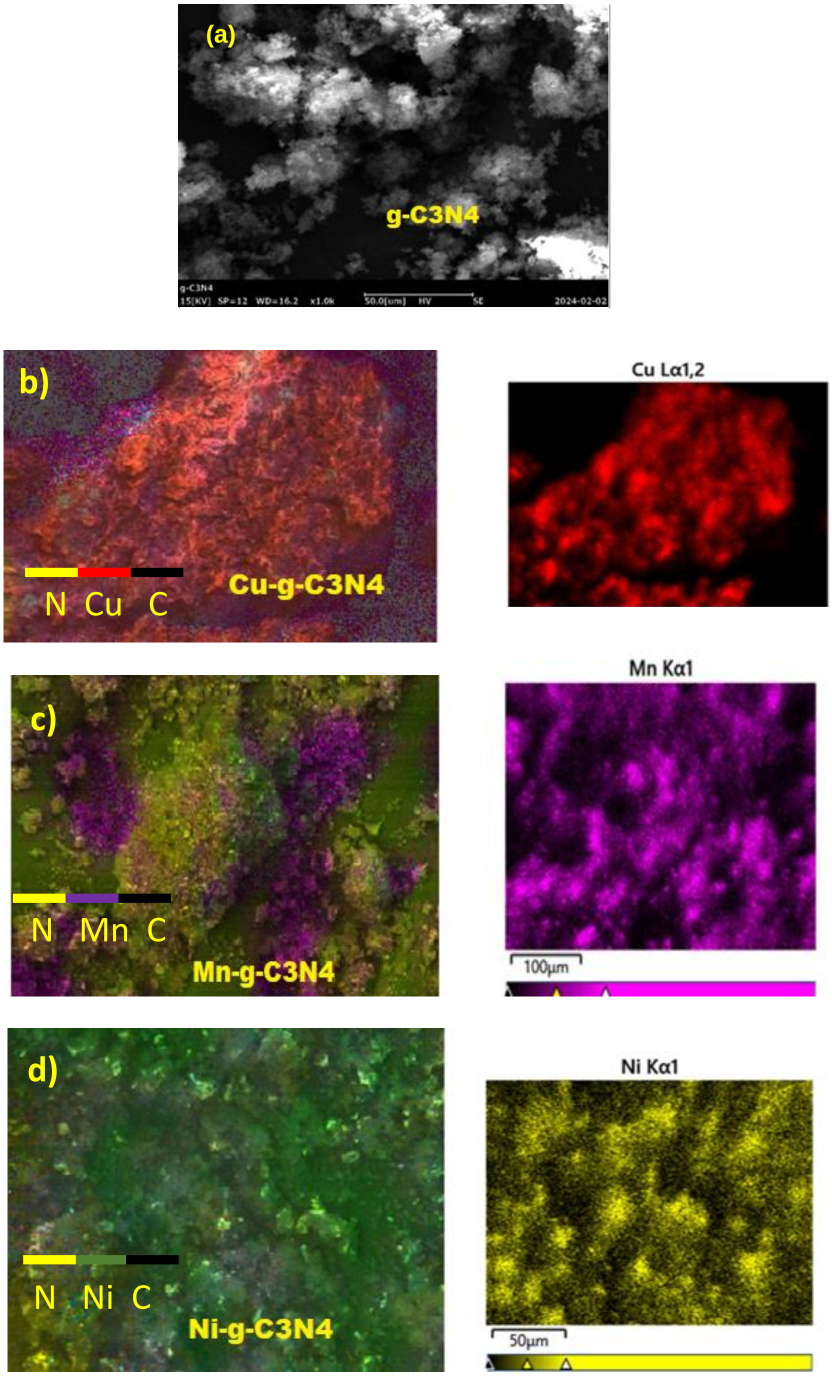

(a)
g-C3N4
g-C3N4
15[KV] SP=12 WD=16.2 x1.0k
50.0[um] HV SE
2024-02-02
b)
N Cu C
Cu-g-C3N4
Cu Lα1,2
c)
N Mn C
Mn-g-C3N4
Mn Kα1
100μm
d)
N Ni C
Ni-g-C3N4
Ni Kα1
50μm

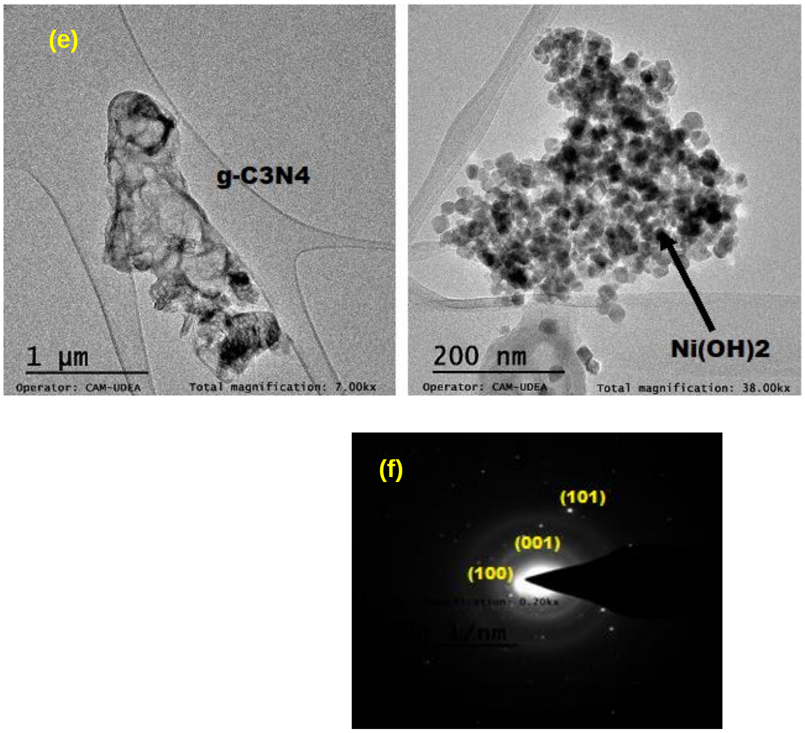

Figure S4. SEM images of (a) g-C_3N_4 (b) Cu-g-C_3N_4, (c) Mn-g-C_3N_4, (d)) Ni-g-C_3N_4, ***(e)*** *TEM images of g-C_3N_4 and Ni-g-C_3N, and (f) diffraction pattern of Ni-g-C_3N_4*

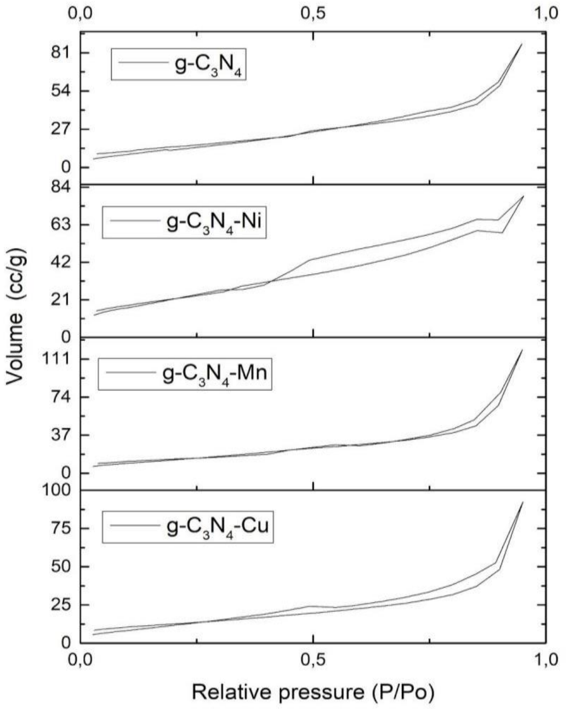

Figure S5. N_2 adsorption-desorption isotherms of g-C_3N_4 and modified materials.

Table S1. Electronic properties and application in 2D- 2D structures and Metal-g-C_3N_4.

Materials	Electronic Properties	Applications
Graphene 2D-2D materials.	Polarization independent tunable bandwidth absorber	Space science and communication science [13].
2D graphene nanosheets-supported semiconductor composite.	Enhancing graphene's electrical conductivity, increasing the number of electrocatalytic active sites on graphene, and strengthening the interface coupling between semiconductors and graphene	Photocatalytic pollutant degradation, H_2 production, and CO_2 reduction [18] [19].
Ni/g-C_3N_4, g- C_3N_4	Ni nanoparticles greatly improve the photocatalytic activity	Degradation of methylene blue and ciprofloxacin, *N*-alkyl pharmaceuticals [20] [21].

		and polycyclic aromatic hydrocarbon [22] [23].
NiO/g-C_3N_4 system	The dominant active species h^+ and $\cdot O_2^-$ from the photocatalysis	Disinfectant properties against E. Coli [24].
Cu-g-C_3N_4/Polyethylene.	Modification in electronic properties for copper coordinated to -N-triazine groups.	Disinfectant properties and methyl blue degradation [25].
Cu Nanoparticles in Cu/g-C_3N_4 Composites.	Double heterojunction	Photocatalytic Reduction of CO_2 to Methanol [26].
Mn/g-C_3N_4/$BiPO_4$	Several reactive species such as h^+, $OH\cdot$ and O_2^- were generated	Degradation of tetracycline (TC) [27].
Metal/g-C_3N_4/ZnO	More efficient separation and conduction of photo-induced charges.	Degradation Methyl orange (MO) and Eriochrome Black T (EBT) dyes [28].

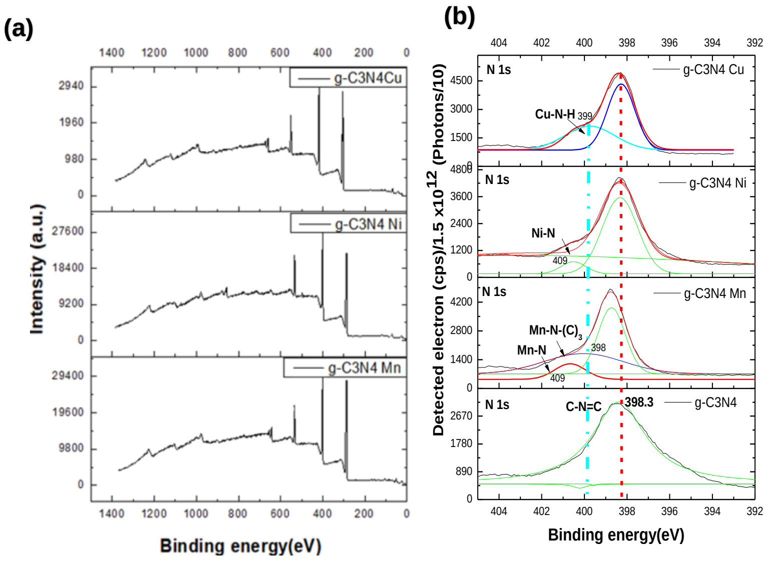

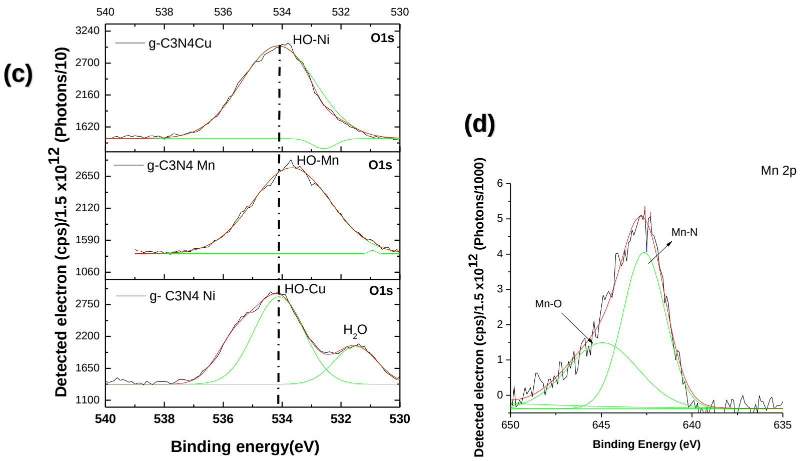

Figure S6. XPS spectra for modified g-C_3N_4 materials, (a) Survey XPS for g-C_3N_4 modifications, (b) High-resolution N 1s orbital contributions, (c) High-resolution O 1s orbital contributions, (d) High-resolution Mn 2p orbital contributions.
